# Early failure of primary repair of a Bochdalek diaphragmatic defect during retroperitoneal liposarcoma resection: a case report

**DOI:** 10.1093/jscr/rjag457

**Published:** 2026-06-13

**Authors:** Takuya Harada, Konomi Takemoto, Naoki Okada, Nozomi Minagawa, Yoshiaki Maeda

**Affiliations:** Department of Gastroenterological Surgery, Hokkaido Cancer Center, 4-2 Kikusui-4-Jo, Shiroishi-ku, Sapporo, Hokkaido 003-0804, Japan; Department of Gastroenterological Surgery, Hokkaido Cancer Center, 4-2 Kikusui-4-Jo, Shiroishi-ku, Sapporo, Hokkaido 003-0804, Japan; Department of Gastroenterological Surgery, Hokkaido Cancer Center, 4-2 Kikusui-4-Jo, Shiroishi-ku, Sapporo, Hokkaido 003-0804, Japan; Department of Gastroenterological Surgery, Hokkaido Cancer Center, 4-2 Kikusui-4-Jo, Shiroishi-ku, Sapporo, Hokkaido 003-0804, Japan; Department of Gastroenterological Surgery, Hokkaido Cancer Center, 4-2 Kikusui-4-Jo, Shiroishi-ku, Sapporo, Hokkaido 003-0804, Japan

**Keywords:** Bochdalek hernia, retroperitoneal liposarcoma, diaphragmatic defect, central tendon, diaphragmatic reconstruction

## Abstract

Adult Bochdalek hernia is a rare congenital diaphragmatic defect that may complicate the surgical management of retroperitoneal tumors extending into the thoracic cavity. An 87-year-old woman presented with a 24-cm retroperitoneal liposarcoma protruding into the thoracic cavity through a Bochdalek defect. En bloc tumor resection with left nephrectomy and partial diaphragmatic resection was performed. The 5-cm diaphragmatic defect appeared suitable for tension-free primary closure and was repaired with interrupted nonabsorbable sutures. However, on postoperative day 5, acute intrathoracic herniation of the stomach occurred. Reoperation revealed enlargement of the defect to 8 cm, and mesh reconstruction was performed. Chronic stretching of the diaphragm and involvement of the central tendon likely compromised tissue integrity and contributed to early repair failure. Mesh reinforcement should be considered when diaphragmatic defects appear structurally weakened.

## Introduction

Bochdalek hernia is a congenital posterolateral diaphragmatic defect caused by incomplete fusion of the pleuroperitoneal membrane during embryonic development. Although it is usually diagnosed in neonates, some cases remain asymptomatic and undetected until adulthood and are occasionally identified incidentally on imaging studies [1].

In adults, diaphragmatic herniation may also occur as a postoperative complication following abdominal or thoracoabdominal surgery. Proposed mechanisms include direct mechanical injury to the diaphragm, thermal injury from energy devices, and excessive traction during organ mobilization [2–4]. These injuries may remain unrecognized intraoperatively and present as delayed diaphragmatic herniation months or years later [2, 4].

In contrast, the management of pre-existing diaphragmatic defects encountered during oncologic surgery has been less frequently discussed. Large retroperitoneal tumors may chronically stretch and attenuate the diaphragm as they extend toward the thoracic cavity. In such situations, diaphragmatic resection and reconstruction may be required, but the optimal repair strategy remains unclear.

Here, we report a rare case of early postoperative diaphragmatic herniation following resection of a giant retroperitoneal liposarcoma associated with a Bochdalek defect, in which primary repair failed in the early postoperative period and required emergency mesh reconstruction.

## Case report

An 87-year-old woman was referred to our department for treatment of right-sided lung cancer. During evaluation, a retroperitoneal tumor that had been followed conservatively at a local clinic was found to have gradually increased in size. After radiotherapy for lung cancer (48 Gy in four fractions), follow-up computed tomography (CT) showed further enlargement of the retroperitoneal mass.

Her medical history included thyroid goiter, and she had been prescribed antiplatelet therapy by her previous physician. Physical examination revealed a palpable mass in the left upper quadrant. Based on imaging findings, the tumor was radiologically suspected to be a dedifferentiated liposarcoma, and surgical resection was planned.

**Figure 1:**
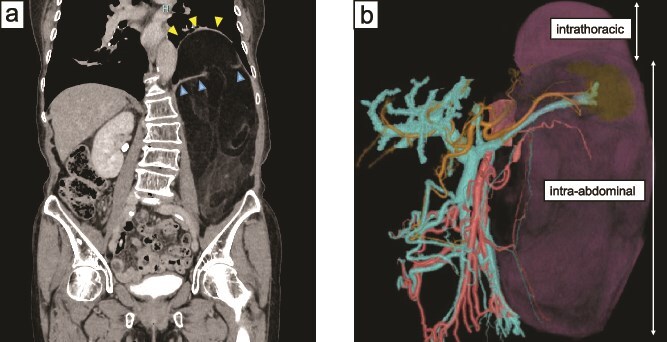
Preoperative imaging findings. (a) Contrast-enhanced CT showing a large retroperitoneal tumor protruding into the left thoracic cavity through a diaphragmatic defect. The diaphragm appears markedly thinned and stretched by the tumor (arrows). This finding suggests chronic mechanical stress on the diaphragm caused by the large tumor. (b) Three-dimensional reconstructed image demonstrating partial intrathoracic extension of the tumor through the diaphragmatic defect.

Contrast-enhanced CT demonstrated a large heterogeneous fat-containing mass measuring ~240 mm in the left retroperitoneal space. The tumor contained multiple soft tissue density nodules and protruded into the thoracic cavity through a diaphragmatic defect consistent with a Bochdalek hernia (Fig. 1a and b).

The patient underwent tumor resection via a midline laparotomy. The tumor originated from the left posterior pararenal space and extended cranially into the thoracic cavity through the diaphragmatic defect. En bloc resection including left nephrectomy and partial resection of the left hemidiaphragm was performed. The diaphragmatic defect, ~5 cm in diameter, appeared suitable for tension-free closure, and primary repair with interrupted nonabsorbable sutures was performed. Operative time was 4 h 56 min with blood loss of 54 mL.

**Figure 2:**
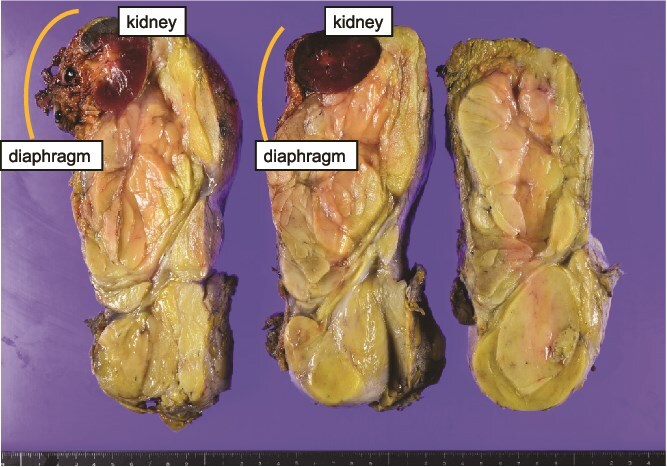
Resected tumor measuring 270 × 220 mm. The specimen includes the left kidney and adrenal gland and shows multiple grayish-white nodules within the fatty tumor. The orange line indicates the resected portion of the diaphragm.

**Figure 3:**
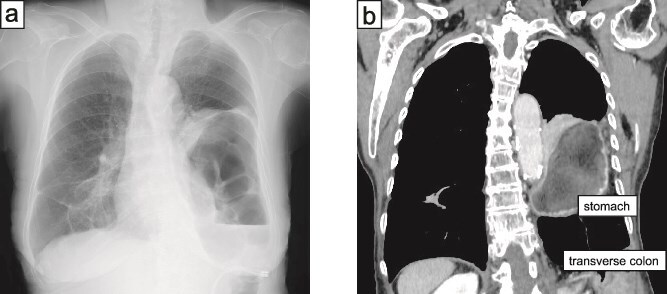
Postoperative imaging findings. (a) Chest radiograph demonstrating elevation of the left hemidiaphragm. (b) Contrast-enhanced CT showing intrathoracic herniation of the stomach (cardia to proximal body) and part of the transverse colon through the diaphragmatic defect.

The resected specimen measured 270 × 220 mm and included the left kidney and adrenal gland (Fig. 2). Histopathological examination showed well-differentiated liposarcoma with focal areas of dedifferentiation. Tumor cells were immunohistochemically positive for MDM2 and CDK4. The early postoperative course was initially uneventful, and oral intake resumed on postoperative day (POD) 3. However, on POD 5, the patient developed vomiting. Chest radiography showed elevation of the left hemidiaphragm (Fig. 3a). CT confirmed intrathoracic herniation of the stomach through the repaired diaphragmatic defect (Fig. 3b).

**Figure 4:**
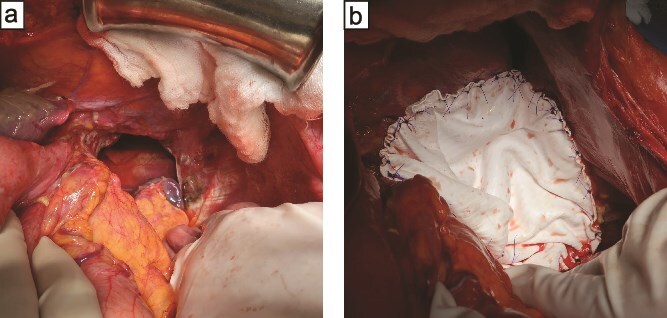
Intraoperative findings at reoperation. (a) Enlarged diaphragmatic defect measuring ~8 cm with direct visualization of the thoracic cavity. (b) Repair of the defect using composite mesh secured to the surrounding diaphragm and vertebral fascia with interrupted nonabsorbable sutures.

Emergency reoperation was performed on the same day. The herniated stomach was viable and easily reduced. The diaphragmatic defect had enlarged to ~8 cm, and the surrounding tissue appeared fragile (Fig. 4a). Primary re-approximation was considered unreliable; therefore, reconstruction using a composite mesh was performed (Fig. 4b). The mesh was secured to the surrounding diaphragm and vertebral fascia with interrupted nonabsorbable sutures.

The second postoperative course was uneventful. The patient resumed oral intake on POD 4 and was discharged on POD 35 after the initial surgery. At 19 months of follow-up, no recurrence of tumor or diaphragmatic hernia has been observed.

## Discussion

Postoperative diaphragmatic hernia is an uncommon but clinically significant complication following abdominal or thoracoabdominal surgery. It has been reported after procedures such as hepatectomy, esophagectomy, and colectomy [2, 3]. In most cases, diaphragmatic defects remain asymptomatic immediately after surgery and become clinically apparent months or years later [2–4].

The present case is unusual because herniation occurred in the early postoperative period after primary repair of a pre-existing diaphragmatic defect. Several factors likely contributed to the failure of the primary repair. First, the defect involved the central tendon of the diaphragm. Unlike the muscular portion of the diaphragm, the central tendon consists primarily of fibrous connective tissue and lacks contractile strength, which may increase the risk of suture pull-through under postoperative intra-abdominal pressure.

Second, the diaphragm had likely been chronically stretched by the large retroperitoneal tumor before surgery. Prolonged mechanical stress may lead to thinning and weakening of the diaphragmatic tissue, reducing its resistance to mechanical load after repair.

Third, patient-related factors such as advanced age and previous radiotherapy may have further compromised tissue quality and wound healing.

During reoperation, the diaphragmatic defect had enlarged and the surrounding tissue appeared fragile, making secure primary closure difficult. Therefore, mesh reconstruction was selected to achieve stable repair. Composite mesh provides mechanical reinforcement while reducing the risk of visceral adhesion and is commonly used for large diaphragmatic defects when primary closure is not feasible [2, 5].

Our institutional experience also provides context for this case. Among 139 retroperitoneal tumor resections performed at our institution, including 14 cases requiring diaphragmatic resection, none developed postoperative diaphragmatic hernia after primary repair. The present case appears exceptional, likely due to the combination of a pre-existing diaphragmatic defect and chronic tumor-induced stretching of the diaphragm.

This case highlights the importance of careful intraoperative assessment of diaphragmatic tissue quality when repairing defects encountered during oncologic surgery. Even when tension-free closure appears possible, diaphragmatic defects involving the central tendon may be particularly vulnerable to early repair failure when the tissue has been chronically stretched by a large retroperitoneal tumor; in such high-risk situations, mesh reinforcement should be considered.

## References

[1] Jones EK, Andrade R, Bhargava A et al. Surgical management of delayed-presentation diaphragm hernia: a single-institution experience. JTCVS Tech 2022;13:263–9. 10.1016/j.xjtc.2022.04.012 35711179 PMC9197083

[2] Raakow J, Megas IF, Schmelzle M et al. Incidence, diagnosis and repair of a diaphragmatic hernia following hepatic surgery: a single center analysis of 3107 consecutive liver resections. J Clin Med. 2021;10:1011. 10.3390/jcm10051011 PMC7958852 33801470

[3] Dell’Abate P, Bertocchi E, Dalla Valle R et al. Iatrogenic diaphragmatic hernia following laparoscopic left colectomy for splenic flexure cancer: an unusual complication. Ann Ital Chir. 2016;5:1–3. 28232645

[4] Shakeel O, Noor MA, Shakeel O et al. Post esophagectomy diaphragmatic hernia (PEDH): an experience of a dedicated cancer center of Pakistan. Clin Oncol Res. 2020;3:1–4. 10.31487/j.COR.2020.08.14

[5] Laila J, Zahra LF, Mustapha O. Diaphragmatic hernia following a liver resection: a rare cause of bowel obstruction. Int J Case Reports Images 2018;9:1. 10.5348/100915Z01NE2018CR

